# Adolescent dietary patterns in Fiji and their relationships with standardized body mass index

**DOI:** 10.1186/1479-5868-10-45

**Published:** 2013-04-09

**Authors:** Jillian T Wate, Wendy Snowdon, Lynne Millar, Melanie Nichols, Helen Mavoa, Ramneek Goundar, Ateca Kama, Boyd Swinburn

**Affiliations:** 1 School of Health and Social Development, Deakin University, Melbourne, Australia; 2 Pacific Research Centre for Prevention of Obesity and Non-communicable Diseases (C-POND), Fiji National University, Suva, Fiji; 3 WHO Collaborating Centre for Obesity Prevention, Deakin University, Melbourne, Australia; 4 British Heart Foundation Health Promotion Research Group, Department of Public Health, University of Oxford, Oxford, UK; 5 Fiji National Food and Nutrition Centre, Suva, Fiji; 6 School of Population Health, University of Auckland, Auckland, New Zealand

**Keywords:** Adolescents, Dietary patterns, Overweight and obesity

## Abstract

**Background:**

Obesity has been increasing in adolescents in Fiji and obesogenic dietary patterns need to be assessed to inform health promotion. The objective of this study was to identify the dietary patterns of adolescents in peri-urban Fiji and determine their relationships with standardized body mass index (BMI-z).

**Methods:**

This study analysed baseline measurements from the Pacific Obesity Prevention In Communities (OPIC) Project. The sample comprised 6,871 adolescents aged 13–18 years from 18 secondary schools on the main island of Viti Levu, Fiji. Adolescents completed a questionnaire that included diet-related variables; height and weight were measured. Descriptive statistics and regression analyses were conducted to examine the associations between dietary patterns and BMI-z, while controlling for confounders and cluster effect by school.

**Results:**

Of the total sample, 24% of adolescents were overweight or obese, with a higher prevalence among Indigenous Fijians and females. Almost all adolescents reported frequent consumption of sugar sweetened beverages (SSB) (90%) and low intake of fruit and vegetables (74%). Over 25% of participants were frequent consumers of takeaways for dinner, and either high fat/salt snacks, or confectionery after school. Nearly one quarter reported irregular breakfast (24%) and lunch (24%) consumption on school days, while fewer adolescents (13%) ate fried foods after school. IndoFijians were more likely than Indigenous Fijians to regularly consume breakfast, but had a high unhealthy SSB and snack consumption.

Regular breakfast (p<0.05), morning snack (p<0.05) and lunch (p<0.05) consumption were significantly associated with lower BMI-z. Consumption of high fat/salt snacks, fried foods and confectionery was lower among participants with higher BMI-z.

**Conclusions:**

This study provides important information about Fijian adolescents’ dietary patterns and associations with BMI-z. Health promotion should target reducing SSB, increasing fruit and vegetables consumption, and increasing regularity of meals among adolescents. Future research is needed to investigate moderator(s) of inverse associations found between BMI-z and consumption of snacks, fried foods and confectionery to assess for potential reverse causality.

## Background

The prevalence of overweight and obesity has increased globally among both adults and adolescents [1,2]. In Fiji, the 2002 National Non-communicable Diseases (NCD) Steps Survey [3] found that amongst those aged 15 to 64 years 29% were overweight and 18% were obese. The prevalence varied by ethnic and sex groups; it was higher for Indigenous Fijian than IndoFijian, and females than males [3]. Among adolescents, data from the 2004 National Nutrition Survey [4] showed that 15% in age groups 10–14 years and 15–17 years were overweight or obese.

As with the adults, more Indigenous Fijians than IndoFijians and females than males were either overweight and obese [3]. Obesity during adolescence increases the risk for metabolic syndrome, diabetes, and cardiovascular diseases, and some forms of cancer [5,6] in adulthood. It is therefore critical to reduce obesity in this age group to arrest the development of non-communicable diseases. There is evidence suggesting the rise in overweight is very steep for older adolescents rising from approximately 15% in the age group 15–17 years to about 47% in the age group 18–24 years [4,7].

Obesity in adolescence has been linked with poor diet and insufficient physical activity [8-10]. The World Health Organization (WHO) has recommended a diet low in fat, sugar and salt, and high in fruit and vegetables in order to protect against the development of obesity [11]. The association between poor diet and obesity is well established globally and regionally [12,13] indicating a low consumption of fruit and vegetables and high consumption of energy-dense food and drinks, along with irregular meal consumption and frequent consumption of SSB and snacks. However, increasing consumption of highly processed food, high fat, salt and sugary foods, and decreasing intake of fruit and vegetables has been documented in many Pacific Island countries [14]. In Fiji, these dietary changes have included a shift towards consumption of energy-dense foods and decreased intake of fruit and vegetables [4,7,15,16]. There has been increased availability of processed food both locally produced and imported, with the urban population increasingly reliant on store bought food. While traditional foods are highly valued in most urban families they are now mainly consumed at special events and family gatherings with most meals instead being based on rice, bread and canned food [4,17]. There is little control over the food supply in schools, including tuck-shop, canteens, and outside vendors who mainly provide energy- dense snacks and drinks.

Studies undertaken in other parts of the world that have examined the association between dietary patterns and weight status in primary school aged children and in adolescents have found that regular meal patterns were associated with lower standardized body mass index (BMI-z) while breakfast skipping, high consumption of snacks, SSB and low consumption of fruit and vegetables were associated with increased BMI-z [18-21]. Such information is needed in Fiji given the escalating problem of obesity among adolescents.

It is also important to consider the ethnic and sex differences in order to inform health promotion interventions. Fiji’s two ethnic groups experience different rates of non-communicable diseases [3] and have substantially different diet and lifestyles [4,7]. A dietary behaviour could be a priority for intervention because it is known to be obesogenic and it has a high frequency in the population. A relatively low frequency dietary behaviour may also be a priority target if it appears to have a major impact on BMI-z. This paper identifies the key dietary behaviours of adolescents in Fiji and their relationship with BMI-z.

## Methods

### Study design

The study analysed data derived from the existing baseline measures (2005/06) obtained from adolescents in schools that were involved in the quasi-experimental intervention study for the ‘Healthy Youth Healthy Communities’ project (the Fiji component of the Pacific Obesity Prevention in Communities (OPIC) Project). OPIC was a community-based obesity prevention study conducted in selected sites in Australia, New Zealand, Tonga and Fiji and aimed to prevent obesity by building community capacity to promote healthy eating and physical activity. Further details of the study design are available in Swinburn et al. [22]. The study was granted ethical approval from the Fiji’s National Health Research Committee and the Fiji National Research Ethics Review Committee and Deakin University Human Ethics Committee, Australia and registered as a trial (ACTRN12608000345381).

### Participants

Participants comprised students aged 13–18 years recruited from 18 secondary schools on the Island of Viti Levu in Fiji. The sample size was 6,871 from the two main ethnic groups in Fiji; Indigenous Fijians and IndoFijians, after excluding 366 ‘other’ ethnic group. The sample comprised 3,271(47.6%; CI 46.4, 48.8) males and 3,600 (52.4%; CI 51.2, 53.6) females with a mean age of 15.6 (SD 1.37) years.

### Measures

#### Sociodemographic characteristics

Students’ ethnicity, age and sex were self-reported. Students were asked which ethnic groups they most identified with. Indigenous Fijians refer to the native Melanesian/Polynesian inhabitants of Fiji [23], and the IndoFijians are Fijians whose ancestors came from various parts of Indian and South East Asia mostly as indentured labourers between 1879 and 1916, but also as free immigrants around 1920s [24,25]. These are definitions used in census surveys in Fiji [26].

#### BMI-z

Anthropometric data (weight and height) were collected by trained research staff using a standardised protocol [22]. Briefly, students were measured using a portable stadiometer (Surgical and Medical PE87) for height to the nearest 0.1 cm and a TANITA Body Composition Analyser (Model BC 418, Wedderburn Australia) for body weight to the nearest 0.1 kg [27]. BMI and BMI-z were calculated based on the WHO categories [28,29] where BMI-z scores over 1 and 2 denote overweight and obesity, respectively.

#### Dietary variables

Students completed a questionnaire about their food and nutrition behaviours, physical activity behaviours and quality of life. This paper reports on the following self-reported behaviours: *Frequency of breakfast, morning snack and lunch consumption,* were assessed with the questions, ‘In the last 5 school days, on how many days did you… [have something to eat for breakfast before school started / eat at morning recess/tea/interval / lunch at lunchtime]?’; *Daily fruit and vegetables consumption* were separately assessed,’ How many serves of [fruit / vegetables] do you usually eat each day?; *SSB consumption* (referring to soft and fizzy, fruit drinks and non-diet drinks) was assessed with four questions: ‘In the last 5 school days (including time spent at home), on how many days did you have regular (non-diet) soft drinks [fruit drinks or cordial (fruit squash or concentrate)] ?’, and ‘On the last school day, how many glasses or cans of soft drinks [fruit drinks or cordial (fruit squash or concentrate)] did you have?’; *Frequency of takeaway consumption for dinner* was assessed with the question ‘How often do you have food from a takeaway shop for dinner?’, and; *Frequent consumption of after school snacks* that were high in fat or high in sugar was assessed with three questions ‘How often do you usually eat biscuits, potato chips or snacks such as instant noodles after school?’, How often do you usually eat pies, takeaways or fried foods such as French fries after school?’ and ‘How often do you usually eat chocolates, lollies, sweets or ice-cream after school? The availability of fruit, potato chips and similar snacks, chocolates and sweets, and non-diet soft drinks at home was also investigated, using the following question structure; ‘How often is/are [food or drink item] available at home for you to eat/drink?

Most of the food and nutrition behaviour questions were either taken directly from or adapted from existing large surveys such as 1995 Australian National Nutrition Survey (NNS) [30], National Children’s Nutrition Survey which was used in New Zealand in 2002 [31] and 1996 Dietary Key Indicators Study [32], and pilot tested with adolescents in Fiji to suit local context [33].

Most questionnaire items provided 4–6 response options and the responses were dichotomised into ‘healthy behaviour’ and ‘less healthy behaviour.’ For example, the variable breakfast consumption was dichotomised into ‘ate breakfast 4–5 days’ and ‘ate breakfast 0–3 days’ in the last 5 school days prior to the survey. Consumption of takeaways and other foods were dichotomised using pragmatic criteria which resulted in different cut-marks due to the likely (and possible) frequency of consumption, and the frequency options that were available in the original questionnaire (which had been refined during pilot testing to ensure they represented the realistic range of consumption frequencies). For instance, while SSBs may be consumed many times per day, the highest possible frequency of ‘consuming take-away for dinner’ would be once per day.The dichotomised dietary variables are detailed in Table 1.

**Table 1:** Dichotomised diet variables

**Diet variable**	**Dichotomised diet variable**	
	**Healthier**	**Less healthy**
**Breakfast, lunch and morning snack**		
Breakfast consumption	Frequent consumer (4–5 days in the last 5 school days)	Infrequent consumer (0–3 days in the last 5 school days)
Source of breakfast	Home	Outside-home (school canteen, shops, friends)
Morning snack consumption	Frequent consumer (4–5 days in the last 5 school days)	Infrequent breakfast consumer (0–3 days in the last 5 school days)
Source of morning snack	Home	Outside-home (school canteen, shops, friends)
Lunch consumption	Frequent consumer (4–5 days in the last 5 school days)	Infrequent lunch consumer (0–3 days in the last 5 school days)
Source of lunch	Home	Outside-home (school canteen, shops, friends)
**Fruit and vegetable**		
Fruit and vegetable consumption	High consumer (≥5 serves a day)	Low consumer (≤5 serves a day)
Fruit consumption after school	Frequent consumer (every day/ almost every day/ most days)	Infrequent consumer (some days/ hardly)
Availability of fruit at home after school	Frequent (every day/ almost every day/ most days)	Infrequent (some days/ hardly)
**Sugar sweetened beverages**		
SSB consumption (frequency)	Infrequent consumer (0–3 days in the last 5 school days)	Frequent consumer (4–5 days in the 5 school days)
SSB consumption (quantity)	Low consumer (≤2 glasses on the last school day)	High consumer (≥ 2 glasses on the last school day)
Availability of SSB at home after school	Infrequent (some days/hardly ever/never)	Frequent (every day/almost every day)
Takeaway consumption	Infrequent consumer (about once a week/2-3 times a month/once a month or less)	Frequent consumer (usually more than once a week)
Takeaway consumption for dinner	Infrequent consumer (2–3 times a month/once a month or less)	Frequent consumer (more than once a week)
**Snacks**		
Buying snacks after school	Infrequent (0–3 days in the last 5 school days)	Frequent (4–5 days in the last 5 school days)
Snacks consumption after school	Infrequent consumer (some days/hardly ever/never)	Frequent consumer (every day/almost every day)
Availability of snacks at home	Infrequent (some days/hardly ever/never)	Frequent (every day/most days) or
Consumption of fried food after school	Infrequent consumer (some days/hardly ever/never	Frequent consumer (every day/most days)
Consumption of confectionary after school	Infrequent consumer (some days/hardly ever/never)	Frequent consumer (every day/most days)
Availability of confectionery at home	Infrequent (some days/hardly ever/never)	Frequent (every day/most days)

### Analysis

Analyses were performed using the statistical software STATA release 11.0 (Stata-Corp., College Station, TX, USA, 2009). The participants’ characteristics and dietary patterns (overall and by ethnicity and sex) were described by cross-tabulations using chi-square tests to determine statistical differences. T-tests were used to assess differences in age, BMI, BMI-z by ethnicity and sex. Linear regression models were used to determine the associations between BMI-z and dietary variables (both overall and stratified by sex and ethnicity), while adjusting for age, clustering effect by school, and sex/ethnicity as appropriate. A test was considered statistically significant if p < 0.05.

## Results

### Descriptive characteristics of participants

**Figure 1:**
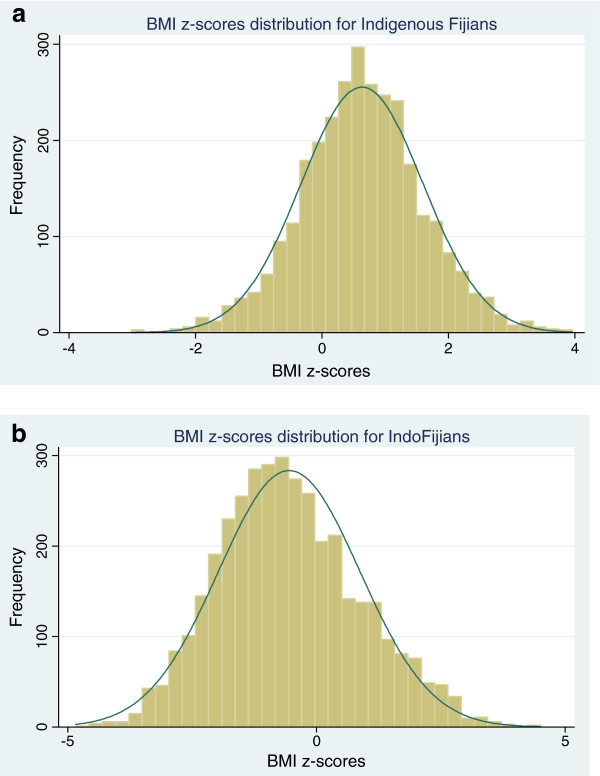
**BMI-z distribution for ethnic sub-groups. a**) BMI-z scores distribution for Indigenous Fijians **b**) BMI z-scores distribution for IndoFijians. The distribution (bars) is not different from the normal distribution curve (lines).

Descriptive statistics are presented in Table 2. Overall, 24% of adolescents were either overweight or obese. Indigenous Fijians were older, taller and heavier than IndoFijians. In addition, despite a similar mean age across sexes, males were heavier, taller and had lower BMI and BMI-z than females. The distribution of BMI-z is shown in Figures 1a and 1b for the Indigenous Fijian and IndoFijian participants respectively. The distribution of BMI-z scores was not significantly different from normal for either ethnic group, however the Indigenous Fijian distribution was shifted to the right (mean = +0.63) compared to the distribution among IndoFijian participants (mean = −0.55).

**Table 2:** Descriptive characteristics of participants

**Characteristics**		**Ethnicity**			**Gender**		
	**Total SD**^**2**^**or 95% CI**^**3**^	**Indigenous Fijian (SD**^**2**^**or 95% CI**^**3**^**)**	**IndoFijian (SD**^**2**^**or 95% CI**^**3**^**)**	**P-value**^**4**^	**Male (SD**^**2**^**or 95% CI**^**3**^**)**	**Female (SD**^**2**^**or 95% CI**^**3**^**)**	**P-value**^**5**^
n	6871	3077	3794		3271	3600	
Age, mean,^1^ years	15.6 (1.37)	15.8 (1.48)	15.4 (1.24)	<0.001	15.6 (1.4)	15.6 (1.4)	NS
Weight, mean, Kg	56.5 (13.8)	63.0 (12.2)	51.2 (12.2)	<0.001	57.9 (14.2)	55.2 (13.2)	<0.001
Height, mean, m	163.2 (8.53)	165.6 (7.83)	161.2 (8.5)	<0.001	167.6 (8.39)	159.1 (6.4)	<0.001
BMI, mean, kg/m^2^	21.1 (4.28)	22.9 (3.75)	19.6 (4.12)	<0.001	20.4 (4.04)	21.7 (4.41)	<0.001
BMI-z scores mean	−0.02 (1.37)	0.63 (0.97)	−0.55 (1.42)	<0.001	−0.21 (1.42)	0.15 (1.31)	<0.001
*Weight status (4 categories)*^*6*^				<0.001			<0.001
Thin (%)	8.3 (7.6, 8.9)	0.45 (0.22, 0.69)	14.5 (13.5, 15.7)		11.4 (10.3, 12.5)	5.44 (4.7,6.2)	
Normal weight (%)	67.9 (66.8,69.0)	65.1 (63.4, 66.9)	70.1 (68.7, 71.6)		69.1 (67.5, 70.7)	66.8 (65.3, 68.7)	
Overweight (%)	17.6 (16.7, 18.5)	26.9 (25.3, 28,4)	10.1 (9.1, 11.0)		13.9 (12.7, 15.1)	21.0 (19.4, 22.3)	
Obese (%)	6.2 (5.7, 6.8)	7.5 (6.6, 8.5)	5.2 (4.5, 5.9)		5.7 (4.9, 6.4)	6.8 (6.0, 7.6)	
*Weight status (2 categories)*^*6*^				<0.001			<0.001
Normal/Thin	76.2 (75.1, 77.20)	65.6 (63.9, 67.3)	84.7 (83.6, 85.9)		80.5 (79.1, 81.8)	72.3 (70.8, 73.7)	
Overweight/Obese	23.8 (22.8, 24.9)	34.4 (32.7, 36.1)	15.3 (14.1, 16.4)		19.5 (18.2, 20.9)	27.7 (26.3, 29.2)	

^1^Means are unadjusted.

^2^SD is standard deviation for means.

^3^ 95% CI for weight status categories.

^4^P-value for the difference in mean and proportion across ethnic sub-groups tested using t-test or Chi-square test, as appropriate.

^5^P-value for the difference in mean and proportion across gender sub-groups tested using t-test or Chi-square test, as appropriate.

^6^ According to WHO classification.

### Dietary patterns of adolescents

#### Meal frequency: breakfast, morning snack and lunch

Adolescents’ dietary patterns are displayed in Table 3. Overall, approximately one-third of adolescents skipped breakfast, morning snack and/or lunch on 2–5 days in the five school days preceding the survey. Compared to IndoFijians, Indigenous Fijians skipped breakfast and morning snack more often. However, for lunch the pattern was reversed, with IndoFijians skipping lunch more often. Regardless of ethnicity, females skipped all three meals more often than males.

**Table 3:** Unadjusted frequency (%) for diet-related behaviours by gender and ethnicity (higher frequency indicates more obesogenic dietary behaviour pattern)

**Dietary variable**	**All**			**Indigenous Fijian (%)**			**IndoFijian (%)**		
	**Total**	**Male**	**Female**	**Male**	**Female**	**Total**	**Male**	**Female**	**Total**^**1**^
	**(95% CI) n= 6,871**	**(95% CI) n=3,271**	**(95% CI) n=3,600**	**(95% CI) n=1,401**	**(95% CI) n=1,676**	**(95% CI) n=3,077**	**(95% CI) n=1,870**	**(95% CI) n=1,924**	**(95% CI) n=3,794**
***Breakfast***									
Infrequent breakfast consumer (0–3 days in the last 5 school days)	23.9	20.3	27.3	26.1	34.1	30.4	16.3	21.8	19.0
	(22.8,25.0)	(18.8,21.8)	(25.7,28.9)*	(23.6,28.7)	(31.5,36.6)*	(28.6,32.2)	(14.5,18.1)	(19.8,23.8)*	(17.7,20.4)*
Breakfast sourced outside from home	4.1	3.9	4.3	5.9	7.2	6.6	2.5	1.9	2.2
	(3.6,4.6)	(3.2,4.6)	(3.6,5.0)	(4.6,7.3)	(5.8,8.6)	(5.6,7.6)	(1.7,3.2)	(1.3,2.6)	(1.7,2.7)*
***Morning snack***									
Infrequent morning snack consumer (0–3 days in the last 5 school days)	35.6	32.7	38.3	39.4	44.9	42.4	28.1	32.9	30.5
	(34.4,36.9)	(30.9,34.4)	(36.6,40.1)*	(36.5,42.3)	(42.3,47.6)*	(40.5,44.4)	(25.9,30.3)	(30.6,35.2)*	(28.9,32.1)*
Morning snack sourced outside from home	62.8	55.9	69.1	71.8	81.1	76.9	45.2	59.4	52.4
	(61.6,64.1)	(54.1,57.8)	(67.4,70.7)*	(69.1,74.4)	(79.0,83.2)*	(75.2,78.6)	(42.8,47.7)	(57.0,61.7)*	(50.7,54.1)*
***Lunch***									
Infrequent lunch consumer (0–3 days in the last 4–5 school days)	23.2	18.3	27.9	17.3	24.3	21.1	18.9	30.9	24.8
	(22.1,24.3)	(16.8,19.7)	(26.3,29.5)*	(15.1,19.6)	(22.0,26.6)*	(19.5,22.8)	(17.0,20.8)	(28.6,33.1)*	(23.4,26.3)*
Lunch sourced outside from home	11.7	9.7	13.6	12.6	18.3	15.7	7.6	9.6	8.6
	(10.9,12.5)	(8.6,10.8)	(12.4,14.8)*	(10.7,14.6)	(16.3,20.4)*	(14.3,17.2)	(6.4,8.9)	(8.2,11.1)*	(7.7,9.6)*
***Fruit and vegetables***									
Did not usually meet recommended fruit & vegetables (less than 5 serves a day)	73.6	71.2	75.7	66.4	70.8	68.8	74.7	79.6	77.1
	(72.5, 74.7)	(69.5,72.9)	(74.2,77.2)*	(63.7,69.2)	(68.4,73.2)*	(67.0,70.6)	(72.3,76.5)	(77.7,81.5)*	(75.6,78.4)*
Infrequent fruit consumed after school (some days or never)	63.1	61.2	64.7	63.2	67.8	65.7	59.9	62.3	61.2
	(61.9,64.3)	(59.5,63.0)	(63.1,66.4)*	(60.4,66.0)	(65.3,70.3)*	(63.9,67.6)	(57.6,62.3)	(60.1,64.6)	(59.5,62.8)*
Unavailability of fruit at home after school (some days or never)	31.5	32.5	30.6	39.6	39.0	39.3	26.9	22.9	24.8
	(30.2,32.7)	(30.7,34.3)	(28.9,32.3)	36.8,42.5)	(36.4,41.6)	(37.4,41.2)	(24.5,29.2)	(20.7,25.0)*	(23.2,26.4)*
***Sugar sweetened beverages (SSB)***									
Frequent SSB consumers (4–5 days in the 5 school days)	89.8 (89.1,90.6)	90.9 (89.8,91.9)	88.9 (87.8,90.0)*	89.3 (87.5,91.1)	89.2 (87.5,90.8)	89.2 (88.0,90.4)	92.0 (90.7,93.3)	88.6 (87.1,90.1)*	90.3 (89.3,91.2)
High consumption of SSB (≥ 2 glasses on the last school day)	70.2	74.6	66.2	77.0	70.0	73.2	73.1	63.2	68.1
	(69.1,71.4)	(73.0,76.2)	(64.6,67.9)*	(74.5,79.4)	(67.6,72.4)*	(71.4,74.9)	(71.0,75.2)	(61.0,65.5)*	(66.5,69.6)*
Frequent availability of soft drink at home after school (every day or almost every day)	33.7	34.2	33.2	21.1	22.0	21.6	44.7	43.6	44.2
	(32.4,35.0)	(32.3,36.0)	(31.4,35.0)	(18.6,23.5)	(19.8,24.3)	(19.9,23.2)	(42.1,47.4)	(41.0,46.2)	(42.3,46.0)*
***Takeaways***									
Frequent food from takeaways (usually more than once a week)	13.2	13.1	13.2	14.7	16.9	15.9	12.0	10.4	11.1
	(12.3,14.0)	(11.9,14.3)	(12.1,14.4)	(12.7,16.8)	(15.0,18.9)	(14.5,17.4)	(10.5,13.6)	(9.0,11.8)	(10.2,12.2)*
Frequent takeaways for dinner (more than once a week)	33.0	33.5	32.5	36.5	36.8	36.7	31.1	28.6	29.8
	(31.7,34.3)	(31.7,35.3)	(30.8,34.3)	(33.7,39.3)	(34.2,39.4)	(34.8,38.6)	(28.7,33.5)	(26.3,31.0)	(28.2,31.5)*
***Snacks***									
Frequent buying of snacks after school ( 4–5 days in the last 5 school days)	22.8	20.0	25.2	23.4	29.5	26.7	17.7	21.9	19.9
	(21.7,23.8)	(18.5,21.5)	(23.7,26.8)*	(20.9,25.8)	(27.1,31.9)*	(25.0,28.4)	(15.9,19.6)	(20.0,23.9)*	(18.6,21.2)*
Frequent snacks consumer (usually after school)	38.3	38.7	37.8	41.2	40.3	40.7	37.1	35.8	36.5
	(37.0, 39.5)	(37.0,40.5)	(36.1,39.5)	(38.4,44.1)	(37.8,42.9)	(38.8,42.7)	(34.8,39.4)	(33.6,38.1)	(34.9,38.1)*
Frequent availability of snacks at home (every day or most days)	50.3	51.9	48.9	52.0	50.7	51.3	51.8	47.2	49.4
	(49.0,51.7)	(50.0,53.8)	(47.1,50.7)*	(49.1,55.0)	(48.1,53.4)	(49.4,53.3)	(49.2,54.4)	(44.6,49.7)*	(47.6,51.2)
Frequent fried foods consumed after school (every day or most days)	12.6	11.8	13.3	10.2	14.0	12.3	12.9	12.7	12.8
	(11.7,13.4)	(10.6,13.0)	(12.1,14.5)	(8.4,12.0)	(12.2,15.8)*	(11.0,13.5)	(11.3,14.5)	(11.2, 14.3)	(11.7,13.9)
Frequent chocolate/sweets consumed after school (every day or most days)	26.2	20.7	31.2	17.8	28.5	23.7	22.7	33.3	28.2
	(25.1,27.4)	(19.2,22.2)	(29.6,32.8)*	(15.6,20.1)	(26.1,30.9)*	(22.0,25.3)	(20.7,24.8)	(31.1,35.6)*	(26.6,29.7)*
Frequent availability of confectionery at home (every day or most days)	29.1	27.4	30.7	17.4	20.1	18.9	35.3	40.5	38.0
	(27.9,30.3)	(25.6,29.1)	(29.0,32.4)*	(15.2,19.7)	(17.9,22.2)	(17.3,20.4)	(32.8,37.9)	(38.0,43.0)*	(36.2,39.7)*

*P-value (<0.05) for the difference in percentages across ethnic and gender subgroups tested using Pearson Chi-square Test. Within column All, asterisk on female show difference within gender. Asterisk on Total ^1^ (%) refers to difference within ethnic subgroups. Within ethnic subgroups, asterisk on female shows difference in percentages between genders.

**Figure 2:**
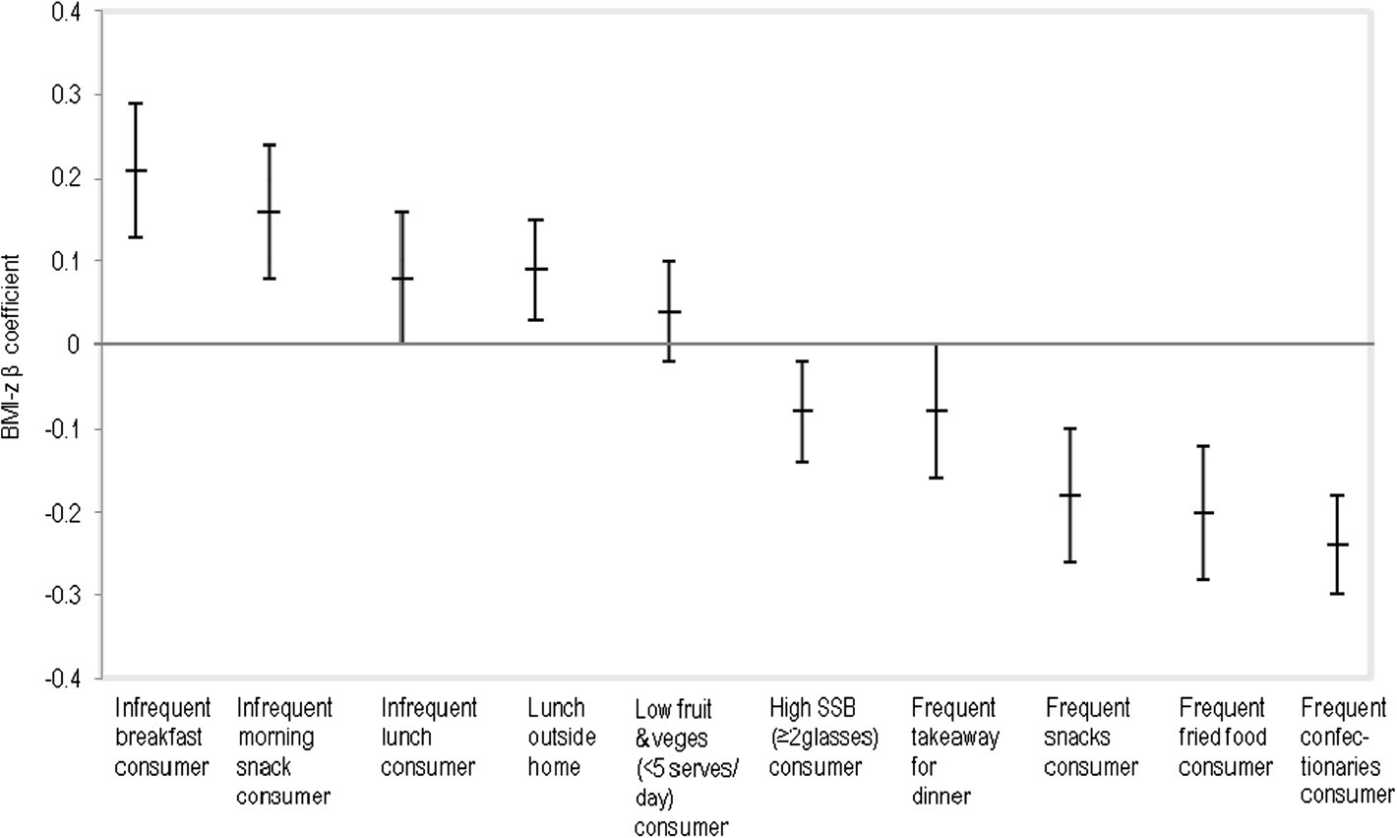
Total sample: adjusted BMI-z ß coefficients for the association between selected dietary variables and BMI-z.

**Figure 3:**
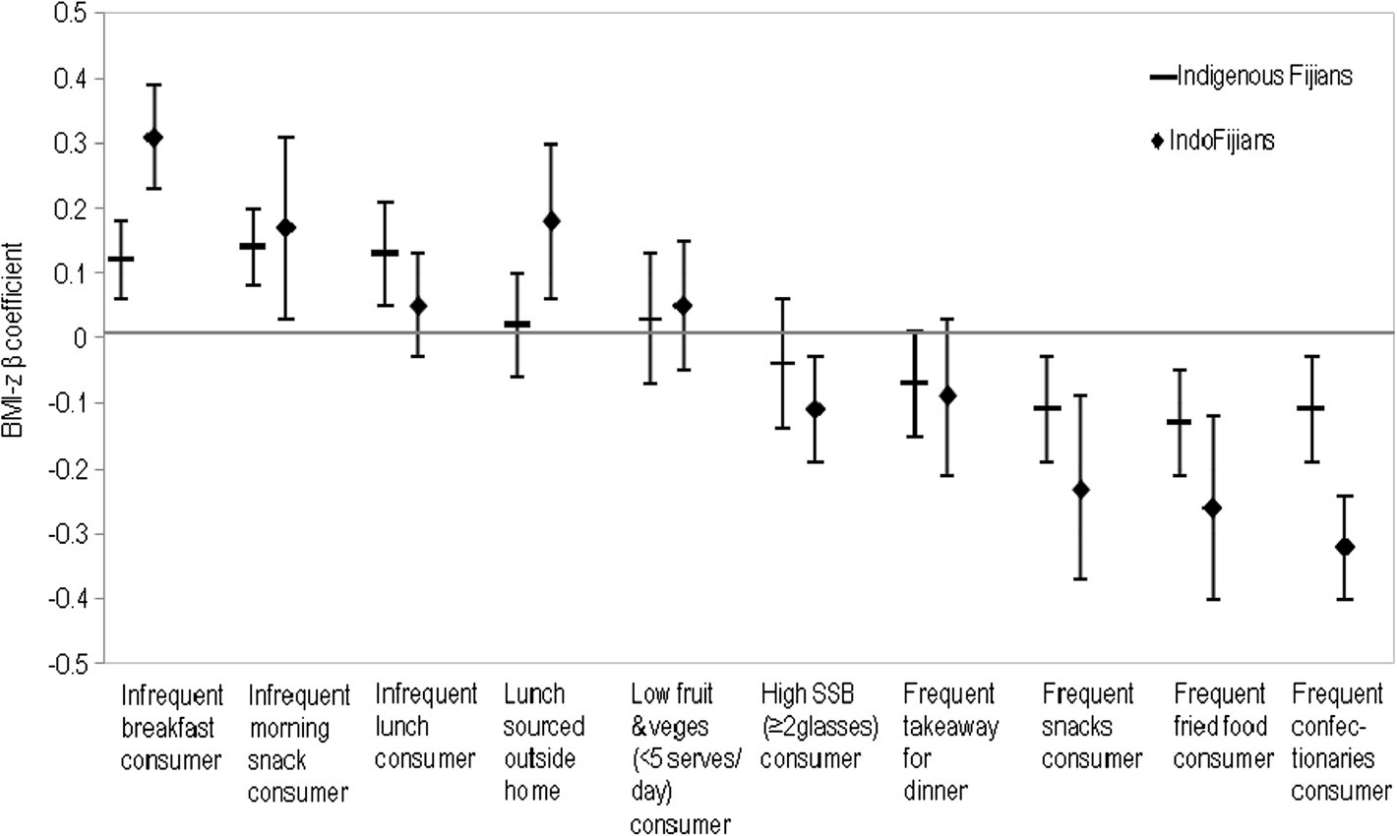
By ethnicity: adjusted BMI-z ß coefficients for the association between selected dietary variables and BMI-z.

**Figure 4:**
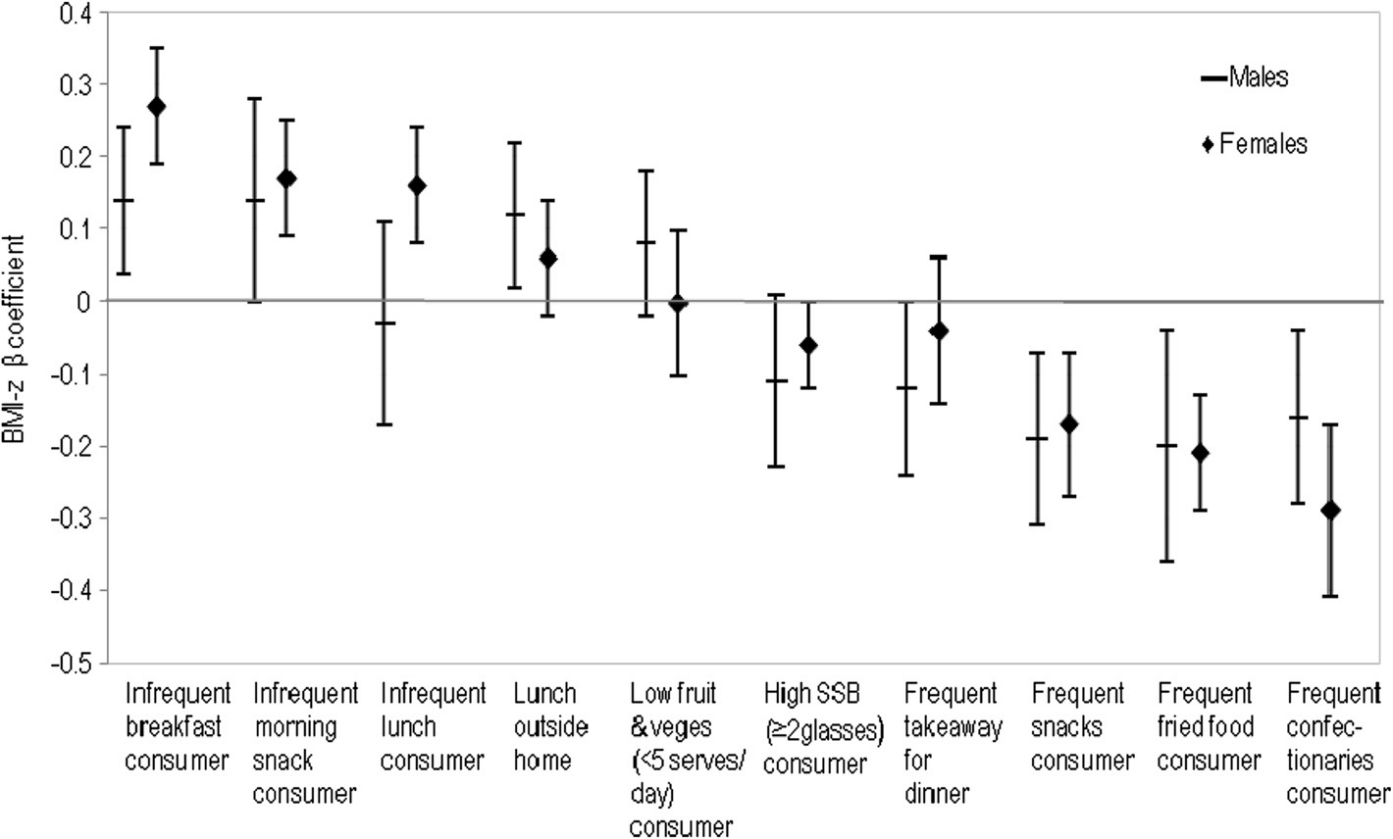
By gender: adjusted BMI-z ß coefficients for the association between selected dietary variables and BMI-z.

Figures 2, 3 and 4 display the association between meal frequency and BMI-z. As expected, irregularity in meals was associated with higher BMI-z ((0.21, p<0.01) and morning snack (0.16, p<0.05)). There was a trend towards a positive association between infrequent lunch consumption and BMI-z but this was not statistically significant. Patterns were similar for both sex and ethnic subgroups.

#### Fruit and vegetables consumption

Overall, nearly three quarters of adolescents failed to meet the recommended ≥5 serves of fruit and vegetables per day [13]; 77% and 69% for IndoFijians and Indigenous Fijians respectively. Similarly, about 71% of males and 76% females failed to meet the recommendation [13]. Figures 2, 3 and 4 show the association between BMI-z consumption and fruit and vegetables with no statistical relationships either overall or by ethnic and sex subgroups.

#### SSB consumption

Almost 90% of adolescents consumed SSB on a regular basis and of those 70% drank two or more glasses on the school day prior to the survey. Overall and in both ethnic subgroups, males consumed more SSB than females. Interestingly, while Indigenous Fijians reported higher consumption of SSB, they were less available in Fijian homes compared to IndoFijian homes.

Figures 2, 3 and 4 show a statistically significant association between SSB consumption and BMI-z, both for the total population and for IndoFijians as a sub group. This association was in an unexpected direction (i.e. higher SSB consumption was associated with lower BMI-z).

#### Consumption of takeaways for dinner

Overall, a third of adolescents ate takeaways for dinner at least once a week. A significantly higher proportion of Indigenous Fijians (37%) than IndoFijians (30%) reported eating takeaways for dinner frequently (more than once a week). The association between consumption of takeaways for dinner and BMI-z was not statistically significant either for the overall population or by sub groups (Figures 2, 3 and 4).

#### After school snacks consumption

Overall, 38% of the adolescents reported eating snacks such as biscuits, potato chips or instant noodles after school, and significantly more Indigenous Fijians (41%) than IndoFijians (37%). Overall, a lower BMI-z was associated with frequent snacking after school. This association was unexpected and held for all the sub-groups.

#### Fried food consumption

Approximately 13% of adolescents reported consuming fried foods after school ‘every day’ or ‘most days’; there were no significant differences by either ethnicity or sex. Unexpectedly, frequent consumption of fried foods after school was associated with a lower BMI-z (−0.19, p<0.001) in the overall sample. This finding was consistent across ethnic and sex sub groups with IndoFijians (−0.26, p <0.05) having lower beta coefficients than Indigenous Fijians (−0.13, p<0.05).

#### Consumption of confectionery

About 26% (CI 25.1; 27.4) of adolescents in the overall sample reported consuming confectionery ‘every day’ or ‘most days.’ Confectionery consumption was significantly higher among IndoFijians (28%) than Indigenous Fijians (24%), as well as females (31%) compared to males (21%). Contrary to expectations, adolescents with lower BMI-z reported more frequent eating of confectionery after school (Figure 2). This was consistent across ethnicity and sex sub groups, with a significantly stronger association among IndoFijians (Figures 3 and 4).

## Discussion

This study aimed to identify some of the key obesogenic dietary patterns of adolescents in Fiji, and associations with BMI-z that could be targeted for obesity prevention. The results highlighted some important ethnic and sex differences in dietary behaviours, as well as some unexpected associations between specific dietary behaviours and BMI-z. This study revealed a high prevalence of overweight or obesity (24% overall), especially amongst Indigenous Fijians (34%) and females (28%). These prevalence figures, which were calculated from data collected in 2005/6, are much higher than the 2004 figures (15%) for a similar age group reported in the National Nutrition report [4]. The 1993 and 2004 National Nutrition data clearly indicated that the proportion of overweight/obese children <18 years in Fiji has more than tripled during this period of time [4,7]. This finding suggests either that the prevalence of overweight or obesity has increased very rapidly, or that the results have been strongly influenced by data collection methods (measured versus self-report) or systematic differences in the sample compositions. The high prevalence and the evidence of increasing trend of overweight or obesity among the adolescents in the current study, highlights the need for serious and targeted health promotion approaches to reduce obesity.

### Meal frequency

While the majority of the adolescents surveyed reported eating regular meals, about a quarter were found to be skipping meals, especially Indigenous Fijians and females. This is a sizeable minority and meal skipping was associated with higher BMI-z. This association is consistent with international evidence [12,20,34,35]. Irregular breakfast consumption was more prevalent among Indigenous Fijians and females. Skipping breakfast was higher in this study than that reported in the United States and Australia [36,37]. A recent study of Indigenous Fijian adolescent females found that those with more westernized values skipped breakfast more commonly than females with traditional values [38]. It can be argued that ‘westernization of food environments’ has placed low value on breakfast consumption. For example, breakfast becomes less relevant because of easy access to energy dense food outside the home. Other studies in Fiji have suggested additional potential reasons for skipping breakfast, including lack of time to prepare and eat breakfast before school [17,39]. Sex differences in meal skipping have been documented in several studies [37,40,41], showing that females skipped breakfast more often than males, and were likely to be either overweight or obese, and this was consistent with the findings of this study.

IndoFijians tended to skip lunch more than Indigenous Fijians. A possible explanation is that among IndoFijians, religious practices such as fasting on some days (males and females) or attending prayer meetings at lunch time (males) are common [42]. Promoting regular healthy meal consumption, particularly breakfast and lunch, should be an important focus for obesity prevention, particularly among females and Indigenous Fijians for both meals, and among IndoFijian males for lunch.

### Fruit and vegetables consumption

Overall, fruit and vegetables consumption was found to be low for the adolescents surveyed compared with adolescents in the United States [43], with only 26% meeting the WHO recommended fruit and vegetables recommendation [13] of 5+ serves a day [44]. However, consumption was higher than found among adolescents in Australia [45]. Low fruit and vegetables consumption was more prevalent among IndoFijians and females in particular suggesting that interventions that aim to increase daily consumption of fruit and vegetables should be prioritised for these sub-groups. Furthermore, a systematic review on this topic by Geller and Dzewaltowski [46], found that a low intake of fruit and vegetables by youth in general worsens with age, thus appropriate age-specific strategies may be needed.

The current study found no statistically significant cross-sectional association between fruit and vegetables consumption and BMI-z. A study by Lin and Morrison [47] indicated that fruit consumption is associated with lower BMI among children while there was no consistent relationship between vegetables intake and BMI. However, independent of the effects on BMI, it is important to target increased fruit and vegetables consumption due to other health benefits such as protection against chronic diseases [48,49]. While future interventions in Fiji need to reach all adolescents, approaches should be tailored to ensure effectiveness among IndoFijians and females who are at most risk of having an inadequate fruit and vegetables intake.

### SSB consumption

In the analysis of the total sample, the consumption of SSB was very high in terms of frequency of consumption for all groups however there was a statistical difference in quantity with higher amounts among Indigenous and males. Possible reasons for the higher consumption of SSB among Indigenous Fijians are the affordability and accessibility of these drinks at school. The high consumption of SSBs among all adolescents is possibly due to availability of spending money. It is common for the majority of Indigenous Fijians to receive $2-5/day as unmonitored spending money [50], which may be used to purchase SSB. The increase in consumption of SSB and access to spending money can be linked to the changes occurring in the food environments. For example, there is easy access to SSB outside of home. In addition, SSB were less available in Indigenous Fijian homes compared to IndoFijian homes (p<0.05). These data suggest that Indigenous Fijians consumed more SSB at school or on the way home.

No significant association was detected between the high intake of SSB and BMI-z. These findings are inconsistent with available evidence that indicates a link between consumption of SSB and excess body weight [51-55]. Despite no relationship being observed, the high prevalence of SSB consumption is of real concern given its lack of nutritional value, association with poor dental health [56], and potential to displace more nutritious foods and drinks in adolescent diets. Health promotion programs need to find effective ways of reducing SSB especially with strategies focused on Indigenous Fijian and male adolescents.

### Takeaways for dinner

About a third of adolescents ate takeaways for dinner more than once a week (termed ‘frequently’ in this study), particularly among Indigenous Fijians. Frequency of takeaway consumption in general was relatively lower in this study than found in other countries [57]. The low prevalence of takeaways for dinner is possibly due to inaccessibility for most households and the prohibitive costs (takeaways in Fiji are often expensive).

No association was found between takeaway consumption for dinner and BMI-z. This result is contrary to Niemeier et al. [58] who found a significant association between relative high intake of takeaways, in this case, restaurant food and obesity. Overall, takeaway consumption for dinner was low in the current study thus it is a lower priority at this time.

### Consumption of snacks after school

Over one third of the study group consumed high fat or salty snacks such as biscuits, potato chips and instant noodles after school, and this was higher among the Indigenous Fijians than IndoFijians. The ethnic differences may be due to differences in parental supervision between the groups, for example picking children up from school. Also many IndoFijian adolescents have reported having dinner (evening meal) soon after they arrived home from school [42], making snacks unnecessary.

Adolescents who consumed more of these high fat or salty snacks had a lower BMI-z. This is an interesting finding, as other studies have showed that increased snacking on these food items is significantly associated with excess body weight among adolescents [18]. This may be due to snacking or eating of other high energy-dense food at other times, desire to lose weight or misreporting of snacking behaviour. Also, the questionnaire did not assess all dietary intakes but assessed only specific foods, which may fail to identify other influential dietary factors. Thus further investigation is needed to understand the moderating factors in such relationship as this information is needed to target health promotion interventions.

### Fried food consumption

Only a minority (10%) of adolescents reported consuming fried foods every day or most days, and this was similar in all subgroups. Frequent intake of fried food was associated with a lower BMI-z. This finding is not supported by number of studies [59-61] which have shown excessive intake of fats in the diet as a significant independent dietary contributor to obesity development. The reasons for this unexpected result are unclear but maybe due to reverse causality or a poor understanding of cooking practices leading to inaccurate responses.

### Consumption of confectionery

Consumption of confectionery every day or most days after school was common among more than 25% of adolescents, particularly the IndoFijians and females. The ethnic difference may be due to IndoFijians having confectionery being more frequently part of the IndoFijians’ cuisine and it being available in their homes after school. The sex difference is possibly due to greater peer influence and cravings among females compared to males [62]. Also, the availability of confectionery at home was higher for females compared to males.

An inverse association was found between frequent consumption of confectionery after school and BMI-z. In particular, IndoFijians and females with lower BMI-z more frequently consumed confectioneries. This finding is inconsistent with available evidence showing positive association between excess body weight and confectionaries [13,63,64] However, Utter [65] has shown adolescents who were trying to lose weight showed this inverse association but it was not seen in those who were not trying to lose weight. This could explain this study’s findings for these adolescents. It is also possible that the findings of this study may be due to reporting bias which has been found among adolescents elsewhere [66,67].

The current study predominantly assessed only frequency of consumption, examining quantity only for SSB. While this provides important information about the prominence of particular key foods and drinks in adolescent diets, and is simple to collect in a short survey from a very large sample such as this, this approach has limitations which may have resulted in some of the weak or unexpected findings in this study. More detailed investigation of these dietary behaviours is needed, including accurate assessment of quantities consumed and the social and environmental context of consumption for Fijian adolescents. This would add further to the evidence base, identifying key targets for health promotion, and may also provide more information to support the development of appropriate and effective strategies. In addition, it was not possible to assess energy expenditure in the current study, which may confound relationships between dietary intake and BMI-z.

### Conclusions and implications

This study has demonstrated that increasing meal regularity (breakfast, morning snack and lunch), decreasing SSB, and increasing fruit and vegetables consumption are likely to be important targets for health promotion in order to encourage a healthful diet for adolescents in Fiji. There were ethnic and sex differences for particular specific behaviours indicating that it is important that obesity prevention interventions are tailored to meet the needs of population sub-groups and that health promotion effort should be tailored accordingly. It is also important to examine in more detail possible reasons for these dietary patterns.

Even though this study did not find a significant association between BMI-z and the consumption of fruit and vegetables and SSB, the significant problems with intake of these items indicate that these behaviours should be the priorities for targeting by health programs, for example provision of healthy choices of food and drinks in school canteens. The inverse association found in this current study between BMI-z and dietary variables such as snacking, eating of fried food and confectionery require further consideration. Future research is needed to investigate moderator(s) of inverse associations found between BMI-z and consumption of snacks, fried foods and confectionery, taking into account the potential for reverse causality (high BMI-z causing adolescents to reduce their intakes of these known obesogenic foods).

## Competing interests

The authors declare that they have no competing interests.

## Authors' contributions

JW developed the objectives of the study as part of her PhD research, performed statistical analysis, interpreted the findings and drafted the manuscript. WS, HM, RG, and AK participated in the interpretation of the findings, and critically edited the manuscript. LM and MN assisted in statistical analysis, supported the interpretation of the findings, and critically reviewed the manuscript. BS participated in the interpretation of the findings and critically reviewed the manuscript. All authors read and approved the final manuscript.
